# Essential oil fractions of hemp profiles at different hydro-distillation periods

**DOI:** 10.1371/journal.pone.0331767

**Published:** 2025-10-29

**Authors:** Valtcho D. Zheljazkov, Vladimir Sikora, Tess Astatkie, Ivayla Dincheva, Milica Acimović, Jelena Visković, Dragana Latković, Jay S. Noller

**Affiliations:** 1 Department of Crop and Soil Science, Oregon State University, Corvallis, Oregon, United States of America; 2 Institute of Field and Vegetable Crops, Novi Sad, Serbia; 3 Faculty of Agriculture, Dalhousie University, Truro, Nova Scotia, Canada; 4 Department of Agrobiotechnologies, Agrobioinstitute, Agricultural Academy, Sofia, Bulgaria; 5 Faculty of Agriculture, University of Novi Sad, Novi Sad, Serbia; 6 Global Hemp Innovation Center, Oregon State University Corvallis, Corvallis, Oregon, United States of America; University of Brescia: Universita degli Studi di Brescia, ITALY

## Abstract

The hypothesis of this study was that hemp (*Cannabis sativa* L.) essential oil (EO) constituents from high-cannabinoid hemp are eluted at different times during the hydro-distillation process, allowing the generation of fractions with distinct chemical profile, with or without cannabinoids. The objective was to reveal changes in the compositional profile of EO fractions captured at ten sequential distillation timeframes (DT) and a control. Regression analysis was conducted to model the relationship between DT and 20 EO compounds, classified in four groups (monoterpenes, sesquiterpenes, cannabinoids, and others (acid esters, ketone, alcohol)) using ten DT values (5, 10, 20, 40, 80, 120, 160, 200, 240, and 280 min). The results showed that most of the EO compounds were eluted early in the distillation process, until 10 min, then the EO compounds gradually decreased until 120 min and decreased to negligible amounts after 160 min DT. Monoterpenes eluted early in the distillation process, accounting for 69.79% of the total EO collected during the 0–5 min distillation interval. After that, the monoterpenes gradually decreased to 13.77% in the 240–280 min DT, while their concentration in the 0–180 min non-stop distillation was 33.55%. Conversely, the sesquiterpenes were eluted late in the distillation process. The concentration of sesquiterpenes in the 0–5 min DT EO was 25.73%, then gradually increased to reach a plateau at 160–280 min DT (75.3–76.8%), while their concentration in the 0–180 min DT was 54.4%. The results demonstrated that hemp EO with higher concentration of monoterpenes and free of cannabinoids can be obtained by separating the initial fractions, while hemp EO with higher concentration of sesquiterpenes and cannabinoids such as cannabidiol (CBD), cannabichromene (CBC), δ8-tetrahydrocannabinol (commonly known as δ8-THC), and δ9-tetrahydrocannabinol (commonly known as δ9-THC or dronabinol) can be obtained by capturing the fraction eluted after 160 min DT.

## 1. Introduction

Hemp (*Cannabis sativa* L.) potential for various applications has garnered attention for its diverse chemical components and their uses [1–3]. One of the primary products derived from hemp is EO, which is predominantly composed of terpenoids [4,5]. The chemical profile of hemp EO is of particular interest due to its organoleptic characteristics, which further influences its utilization and biological activity [6–8]. According to numerous reports, the dominant constituents of hemp EO are mainly sesquiterpenes (including *β*-caryophyllene, *α*-humulene, and caryophyllene oxide), followed by a small portion of monoterpenes (mainly *α*-pinene and *α*-terpineol) [8–10]. However, previous studies indicated that hemp EO profile is influenced by factors such as phenological stages [11,12], harvest time, genetic and environmental influences [13–17], drying methods [18,19], seasonal variations [20], and extraction method [21–23], as well as terms and conditions during extraction, which can significantly influence the quantity and quality of the obtained EO. Therefore, optimizing the extraction process is crucial for obtaining high-value products that can be utilized in multiple applications, i.e., a single raw material to generate products suitable for various industries [24].

Additionally, previous research has demonstrated that grinding, extraction and distillation times can significantly alter the EO profiles of various EO crops [7,25,26], suggesting the potential to produce EO with distinctive characteristics valuable to various industries [5,27,28]. However, the previous research was conducted with industrial hemp (grain and fiber) hemp varieties [16,29,30]. In Europe, most research on hemp EO has focused on varieties approved and listed by the European Union [27,31,32]. These hemp varieties were primarily developed for grain and fiber production, resulting in a relatively low EO content, ranging from 0.001% to 0.1% on a dry weight basis.

Conversely, hemp production and interest in the U.S. and worldwide have centered on varieties developed for cannabinoids production due to interest in its benefits in human health [33–35]. These cannabinoid-type hemp varieties, initially derived from illicit marijuana breeding in the U.S. over the past few decades, are characterized by high cannabinoid and EO content. While these new hemp varieties have been widely cultivated in the U.S., they have not yet been widely adopted in Europe.

It would be important to find out if cannabinoids-rich hemp developed in the U.S. would be successfully grown in the European countries and produce desirable EO profile for the burgeoning hemp EO markets worldwide. Furthermore, it is not known how distillation time of U.S. hemp varieties grown in Europe would affect EO content and composition of such varieties. Therefore, this study hypothesized that EO constituents are eluted at different times during the distillation process, allowing the identification of fractions with desirable compositional profile. The objective was to reveal significant changes in the compositional profile of EO fractions captured at ten sequential distillation timeframes (DT) in comparison to control in terms of EO chemical profile.

Adjusting the DT of hemp can produce EOs of varying quality for different industries. Shorter DT yield monoterpene-rich oils, valued in fragrance, food, and aromatherapy, while longer DT release sesquiterpenes, more suitable for the pharmaceutical industry. Tailoring DT can reduce costs and guide pricing based on chemical composition. Therefore, the main goal of this research is to monitor the quality of hemp EO over the following time frames: 0–5, 5–10, 10–20, 20–40, 40–80, 80–120, 120–160, 160–200, 200–240, and 240–280 min, as well as a standard continuous DT of 3 hours (i.e., 0–180 min), which was used as the control.

## 2. Materials and methods

### 2.1. Plant material, growing conditions, and sampling

The cannabinoids-hemp (*Cannabis sativa* L.) cv. Bob-1 was grown at the Alternative Crops and Organic Production Department in Backi Petrovac (45.336500°N 19.671355°E), a research unit of the Institute for Field and Vegetable Crops in Novi Sad, Serbia. Certified seeds of this variety were generously donated in May 2018 by a commercial hemp producer in Madras, Oregon, U.S.A. The seeds were sown directly into the ground at the end of March, aligning with the typical seeding time for industrial hemp varieties at this latitude and in this region. The cultivar Bob-1, not an officially approved variety, is not listed in the European List of Approved Hemp Varieties [36]. The hemp plants were grown on alluvial chernozem soil with a pH of 7.2 and without irrigation. Traditional industrial hemp varieties cultivated for grain and fiber are usually grown as rainfed crops in most of northern, central, and southeastern Europe. These varieties are typically planted early, between March and April. Thanks to its deep, fast-growing root system, hemp efficiently utilizes the moisture accumulated in the soil over the winter. At the flowering stage on June 20th, the top 50 cm of the hemp plants were harvested, and EO was extracted from the fresh biomass. The harvested material from five adjacent plants was homogenized, and multiple subsamples were prepared for the subsequent distillation study.

### 2.2. Essential oil (EO) extraction

The hemp EO was extracted using hydro-distillation in 4-L hydro-distillation units with most parts of the units resembling Clevenger apparatus, with three replicates. Each sample comprised 300 g of fresh biomass, specifically from the top 50 cm of the plants, including leaves and flowers, with stems removed. The biomass was immersed in 1.5 L of water and distilled to obtain the EO.

All samples underwent continuous distillation. Once the distillation process was complete, the heat source was removed, and the EO was measured by volume using the graduated section of the apparatus. The EO, along with some water, was then collected in glass vials and stored in a freezer. After completing all distillations, the EO was carefully separated from the water, weighed on an analytical scale, and returned to the freezer for storage until gas chromatography (GC) analysis. Therefore, the results on EO yields here are reported on a fresh weight basis. The EO yield is reported here as the volume-to-weight ratio based on the fresh hemp material.

Hemp EO fractions were captured at ten sequential DT (0–5 min, 5–10 min, 10–20 min, 20–40 min, 40–80 min, 80–120 min, 120–160 min, 160–200 min, 200–240 min, and 240–280 min) and a control (0–180 min). In brief, we chose 0–180 min as the control because the standard distillation time for hemp EO is stated to be 3 hours [37]. On the other hand, monoterpenes have a short extraction time, while an extended extraction period is crucial for isolating sesquiterpenes, as their release occurs over a prolonged timeframe (up to 280 min). Therefore, we set the fractionation of volatile components within the DT framework as the focus of this research.

### 2.3. Hemp essential oils (EOs) GC analyses

The composition of hemp EOs was analyzed using a 7890A gas chromatograph (Agilent Technologies Inc., Santa Clara, CA, USA) connected to a 5975C mass selective detector (MSD). A HP-5ms silica fused capillary column (30 m long, 0.32 mm internal diameter, and 0.25 µm film thickness) was used for the analysis. The oven temperature started at 40 °C (with no hold), increased at a rate of 5 °C/min until reaching 300 °C, where it was held for 10 minutes. Helium was the carrier gas with a flow rate of 1.0 mL/min. The injection volume was 1.0 µL and a split ratio of 20:1. The ionization source, quadrupole, and injector temperatures were set at 230 °C, 150 °C, and 250 °C, respectively. The MSD operated in full scan mode, with mass spectra recorded at 70 eV in electron ionization (EI) mode. The EO components were identified by comparing their linear retention indices (LRI) and mass spectral fragmentation patterns with those in the NIST’08 and Adams mass spectra libraries. LRIs were calculated using a mixture of aliphatic hydrocarbons (C_8_ to C_40_) under the same chromatographic conditions.

Additionally, gas chromatography with flame ionization detection (GC-FID) was performed using the same 7890A gas chromatograph equipped with an FID. The HP-5 silica fused capillary column and the oven temperature program were the same as with the MSD analysis. The detector and injector temperatures were set at 280 °C and 220 °C, respectively. The EO samples (1.0 µL) were injected in split mode (20:1), and their percentage composition was determined using the peak normalization method.

### 2.4. Statistical analyses

One-way Analysis of Variance (ANOVA) was completed to determine if there is a significant difference among the ten sequential DT (0–5 min, 5–10 min, 10–20 min, 20–40 min, 40–80 min, 80–120 min, 120–160 min, 160–200 min, 200–240 min, and 240–280 min) and control (0–180 min) in terms of EO, 28 constituents (*α*-pinene, *β*-pinene, myrcene, limonene, *β*-(E)-ocimene, *α*-terpineol, *β*-caryophyllene, *α*-humulene, *δ*-selinene, *γ*-cadinene, *δ*-cadinene, nerolidol, maaliol, germacrene D-4-ol, spathulenol, caryophyllene oxide, globulol, ledol, *γ*-eudesmol, caryophylla-4(12),8(13)-dien-5*α*-ol, *β*-eudesmol, *α*-eudesmol, bulnesol, *α*-bisabolol, cannabidiol (CBD), cannabichromene, δ8-tetrahydrocannabinol (THC), and δ9-tetrahydrocannabinol (dronabinol)), classified into 4 groups (monoterpenes, sesquiterpenes, cannabinoids, and others (acid asters, ketone, alkochol)).

The analyses were completed using the Mixed Procedure of SAS [38]. Since the effect of Fraction was significant (P-value < 0.05) on all responses, further multiple means comparison was completed using Tukey’s multiple range test at 5% level of significance, and letter groupings were generated. For each response variable, the validity of model assumptions was verified by examining the residuals as described in Montgomery [39].

### 2.5. Regression analysis to describe the relationship and to forecast values

Regression analysis was conducted to model the relationship between distillation timeframe (DT) and EO, 22 constituents classified into four groups: monoterpenes (*α*-pinene, *β*-pinene, myrcene, limonene, *β*-(E)-ocimene), sesquiterpenes (*α*-humulene, *δ*-selinene, *γ*-cadinene, *δ*-cadinene, nerolidol, *β*-caryophyllene, maaliol, germacrene D-4-ol, spathulenol, caryophyllene oxide, globulol, *γ*-eudesmol, *α*-eudesmol, caryophylla-4(12),8(13)-dien-5*α*-ol, *β*-eudesmol, bulnesol, and *α*-bisabolol), cannabinoids (CBD, THC and dronabinol), (and others (acid asters, ketone, alkochole), using ten DT values (5, 10, 20, 40, 80, 120, 160, 200, 240, and 280 min). The most appropriate model was either a second-order polynomial (Eq 1), third-order polynomial (Eq 2), Exponential decay (Eq 3), or asymptotic (concave) (Eq 4). The model used for each constituent is indicated in the caption of each Figure.


$$Y=\beta_{\text{0}}+\beta_{\text{1}}X+\beta_{\text{2}}X^{\text{2}}+\varepsilon$$ (1)

$$Y=\beta_{\text{0}}+\beta_{\text{1}}X+\beta_{\text{2}}X^{\text{2}}+\beta_{\text{3}}X^{\text{3}}+\varepsilon$$ (2)

$$Y=\theta_{\text{1}}e^{\theta_{\text{2}}X}+\varepsilon$$ (3)

$$Y=\theta_{\text{1}}-\theta_{\text{2}}e^{-\theta_{\text{3}}X}+\varepsilon$$ (4)

Where Y is the dependent (response) variable, X is the independent (DT) variable, and ε is the error term, which is assumed to have a normal distribution with constant variance.

While the second-order and the third-order polynomial models (Eqs 1 and 2) are linear, the other two models (Exponential decay (Eq 3), and asymptotic (concave) (Eq 4)) are nonlinear and their parameters were estimated iteratively using the NLIN Procedure of SAS [38], and the fitted models met all adequacy requirements of nonlinear models [40]. The figures as well as the second-order and third-order polynomial model fits were completed using Minitab 21 software (Minitab, State College, PA, USA).

Informed Consent: This study did not involve human participants; therefore, informed consent was not required.

## 3. Results and discussion

### 3.1. Essential oil (EO) yields during distillation times (DT)

**Fig 1:**
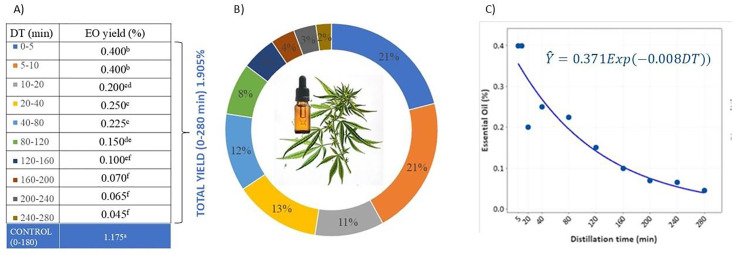
(A) Distillation time (DT) and EO yield, (B) proportion of EO yield in the different DTs, and (C) Plot of DT vs EO along the fitted Exponential decay regression model.

The yield of hemp EO after 280 min of DT was 1.905%, while in the control (0–180 min) it was 1.175% (Fig 1A). In the first 10 min of DT, 42% of the total oil yield was obtained, while extending the DT for another 10 min increases the yield by 11%, which means that practically more than half of the yield (53%) is extracted in the first 20 min of distillation (Fig 1B). As the DT increased from 0–5 min to 240–280 min, there was a general decline in EO yield. This is shown in Fig 1C as a plot of DT vs. EO (%) along with the fitted Exponential decay regression model ($\hat{Y}=\text{0}.\text{3}\text{7}\text{1}e^{-\text{0}.\text{0}\text{0}\text{8}DT}$). This result also shows that most of the EO eluted early in the distillation process, until 10 min, then gradually decreased (0.2% in the DT 10–20 and 20–40) until 120 min and further decreased to negligible amounts (< 0.1%) after 160 min DT. In brief, this study indicates that the majority of hemp EO is extracted early in the distillation process, with extended DT yielding diminishing returns. A similar trend is noted in the EO of turmeric leaves [41], Rocky Mountain juniper leaves [42], residual red ginger waste [43], and immortelle [44].

According to the literature, EO yield in hemp ranges between 0.01 and 0.61%, while the specific yield and composition can vary due to multiple factors related to growing conditions, agro-technique, and postharvest processing [45]. Previous studies have demonstrated that the optimal DT for hydro-distillation of hemp EO is 180 min [22,46,47]. This duration is reported to produce a superior-quality hemp EO, characterized by a sesquiterpene-dominant profile with a notable presence of monoterpenes [48]. Shorter DTs, however, result in EO with a higher yield of monoterpenes, which are valued for their superior olfactory profile, making them more desirable for the fragrance industry [44].

### 3.2. Concentration (yields) of monoterpenes in hemp EO depends on distillation time (DT)

**Fig 2:**
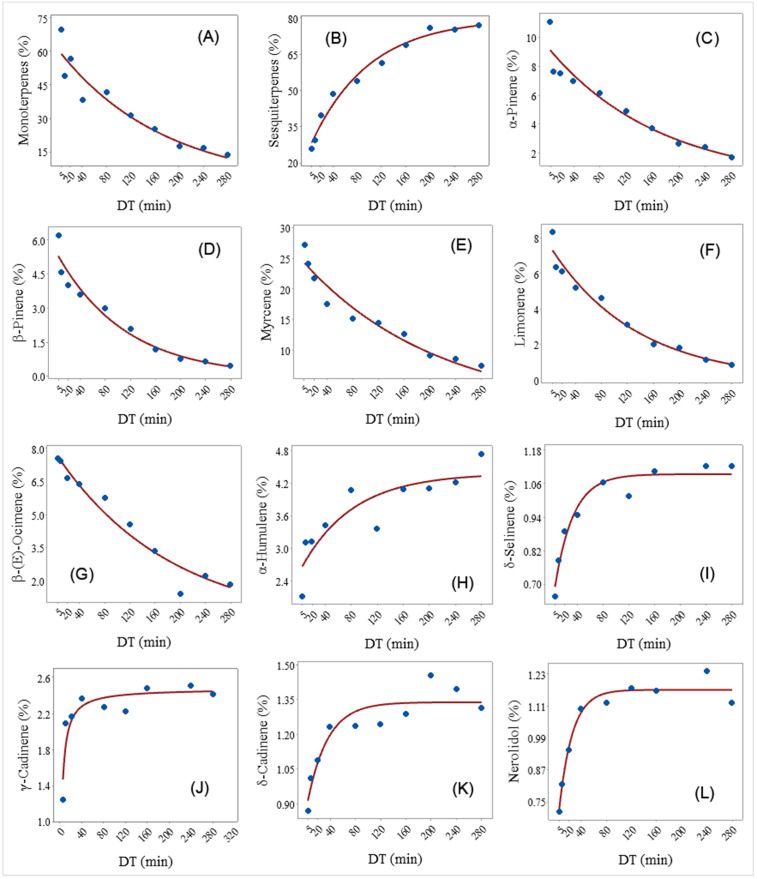
A fitted regression model that shows the relationship between distillation time (DT) and the concentration (%) of (A) Monoterpenes, (B) Sesquiterpenes, (C) α-Pinene, (D) β-Pinene, (E) Myrcene, (F) Limonene, (G) β-(E)-Ocimene (%), and (H) α-humulene, (I) δ-Selinene, (J) γ-Cadinene, (K) δ-Cadinene, and (L) nerolidol.

Monoterpenes (C_10_ compounds) represent a large group of biologically active compounds that consists of two linked isoprene units in basic structure. The monoterpene profile in this research (*α*-pinene, *β*-pinene, myrcene, limonene, *β*-(E)-ocimene, *α*-terpineol) aligns with previously reported data on the EOs of various hemp accessions in the literature [5,8,25,29,37]. The DT vs. the yield of monoterpenes (%) relationship was best described by an Exponential decay regression model ($\hat{\;Y}=\text{6}\text{0}.\text{6}e^{-\text{0}.\text{0}\text{0}\text{5}\text{7}DT}$) (Fig 2A). As expected, the monoterpenes were eluted early in the distillation process and comprised 69.79% of the EO in the 0–5 min DT (Table 1). After that, the monoterpenes in the fractions gradually decreased to 13.77% in the 240–280 min DT, while their yield in the 0–180 min non-stop distillation was 33.55%. Conversely, the sesquiterpenes increased with the increase in DT (Fig 2B).

**Table 1:** Mean concentrations of sum monoterpenes and single monoterpenes: α-pinene, β-pinene, myrcene, limonene, β-(E)-ocimene, and α-terpineol obtained from the 10 distillation time (DT) frames and control (0-180 min) of top hemp plants. $\sqrt{MSE}=$ Square root of Mean Squares Error that estimates the common standard deviation (σ). Within each row, means sharing the same letter are not significantly different.

RI calc	RI lit	DT (min)	0-5	5-10	10-20	20-40	40-80	80-120	120-160	160-200	200-240	240-280	0-180	$\sqrt{MSE}$
		**Monoterpenes**	69.79 a	49.15 bc	56.63 b	38.21 de	41.63 cd	31.51 ef	25.30 fg	17.33 gh	16.64 gh	13.77 h	33.55 def	
**930**	**932**	**α-Pinene**	11.07 a	7.66 b	7.51 b	6.99 bc	6.18 c	4.94 d	3.74 ef	2.68 fg	2.45 g	1.72 g	4.11 de	0.284
**975**	**974**	**β-Pinene**	6.21 a	4.59 b	4.03 c	3.61 c	3.00 d	2.11 e	1.19 f	0.76 fg	0.66 g	0.46 g	2.20 e	0.132
**988**	**988**	**β-Myrcene**	27.28 a	24.18 b	21.73 c	17.53 d	15.13 e	14.52 e	12.57 f	9.15 g	8.51 g	7.40 g	11.94 f	0.324
**1023**	**1024**	**Limonene**	8.32 a	6.33 b	6.12 bc	5.19 cd	4.60 de	3.13 f	2.03 g	1.86 gh	1.17 gh	0.90 h	4.06 ef	0.262
**1042**	**1044**	**β-(E)-Ocimene**	7.56 a	7.46 ab	6.67 bc	6.40 cd	5.78 de	4.56 f	3.36 g	1.41 i	2.23 h	1.83 hi	5.46 e	0.204
**1185**	**1186**	**α-Terpineol**	2.69 de	3.73 ab	3.85 ab	3.46 bc	4.16 a	2.99 cd	2.18 e	1.28 f	1.47 f	1.47 f	2.67 de	0.174

Previous studies have shown that monoterpenes are dominant in fresh plant material but tend to decrease during drying and storage, resulting in a relatively higher yield of sesquiterpenes in dried hemp [19]. Given that the EO in this study was extracted from fresh material, the pronounced presence of monoterpenes in the early distillation fractions (0–5 min) is consistent and expected.

Other research also indicates that monoterpenes like myrcene, ocimene, limonene, α-pinene, β-pinene, and terpinolene are the predominant components of hemp EO, playing a key role in its aroma and biological effects [48,49]. Monoterpenes occur in higher yield at the beginning of the distillation process, as it was previously reported for juniper, rosemary, lavender [50–52], and many other EO-bearing plants. The major monoterpenes in hemp EO in this study were β-myrcene (whose yield varied from 7.40 to 27.28% depend on DT frame), *α*-pinene (1.72–11.07%), *β*-pinene (0.46–6.21%), limonene (0.90–8.32%), *β*-(E)-ocimene (1.41–7.56%), and *α*-terpineol (1.28–4.16%), which is in line with previous publications [48,53,54].

***α*-Pinene** is one of the lowest boiling of all monoterpenes. It is an important flavor compound with characteristic herbal odor (with turpentine and woody scent), diverse biological activities, among them antioxidant, antitumor, antimicrobial and insecticidal [55,56], which makes it highly relevant in everyday life. The relationship between DT and the concentration of *α*-pinene (%) was described by an exponential decay regression model ($\hat{\;Y}=\text{9}.\text{4}e^{-\text{0}.\text{0}\text{0}\text{6}DT}$) (Fig 2C). *α*-Pinene was eluted early in the DT, resulting in 11.07% of total oil in the 0–5 DT, with a significant decrease as DT increased, and reached 1.72% at the 240–280 DT. *α*-Pinene concentration in the control (non-stop distillation 0–180 min) was 4.11%, similar to the one in the 80–120 and 120–160 DT (Table 1). For maximal extraction of *α*-pinene in hemp EO it could be recommend DT between 120 and 160 min. A similar trend, i.e., *α*-pinene yields decreasing with increasing distillation time, was reported previously in rosemary, where the maximal concentration of *α*-pinene in EO is achieved between 120 and 180 min DT [57].

Parallel as *α*-pinene, ***β*-pinene**, as its isomer, also possesses low boiling point, characteristic herbal scent (pine-like woody odor), and wide range of biological properties, especially potent antimicrobial and anticancer activities [58–60]. The relationship between the DT and the concentration of *β*-pinene was described by an exponential decay regression model ($\hat{Y}=\text{5}.\text{5}\text{4}e^{-\text{0}.\text{0}\text{0}\text{9}DT}$). The concentration of *β*-pinene followed a similar trend to that of *α*-pinene, with concentrations decreasing as DT increased ($\hat{Y}=\text{5}.\text{5}\text{4}e^{-\text{0}.\text{0}\text{0}\text{9}DT}$) (Fig 2D). In summary, the highest concentration of *β*-pinene is noted at the beginning of the distillation process (6.21% at 0–5 min), then significantly decreases at 5–10 min, and this trend continuously progresses with the DT (4.03% and 3.61%, at 10–20 min and 20–40 min, respectively). In the control, its concentration is significantly lower, 2.20%, while in the final DT (240–280 min) its concentration is only 0.46% (Table 1). Previous research demonstrated that the highest concentrations of *β*-pinene in the EO were obtained during the 5–10 min distillation of ground fresh hemp biomass. In contrast, the elution dynamics in non-ground material differed significantly, with peak *β*-pinene elution occurring between 80 and 120 minutes [26].

**Myrcene** is commonly used fragrance ingredient in food and beverage industry, cosmetic and household products [61,62]. Apart from its spicy odor type, it is valuable because of its promising health benefits such as antioxidant, anti-inflammatory, antiproliferative antibacterial and analgesic properties [63]. Practically, myrcene is the most dominant monoterpene in hemp EO in this study (11.94% in control), and one of the most common terpenes found in hemp [64]. The concentration of myrcene decreases over time during a distillation process, according to its exponential decay regression model ($\hat{Y}=\text{2}\text{4}.\text{8}\text{4}e^{-\text{0}.\text{0}\text{0}\text{5}DT}$), i.e., concentration of myrcene decreased as DT increased (Fig 2E). The concentration of myrcene was 27.3% in the 0–5 min DT EO and decreased to 7.4% in the 240–280 min DT EO (Table 1). A similar trend was observed in oregano EO, where the myrcene concentration was 6.06% at the beginning of distillation and gradually declined with longer distillation durations, stabilizing between 0.58% and 0.73% from 40 to 360 minutes [65].

**Limonene** is widely used as flavor and fragrance compound in foodstuffs and beverages, cosmetic and household products [66]. It provides a lemon-like odor to the many EOs, among them in hemp [67]. Limonene has significant antimicrobial, anticancer, analgesic, immune regulation, neuroprotection, antioxidant, anti-inflammatory properties and many other pharmacological effects [68]. The concentration of these compounds in the control EO (0–180 min) was 4.06%. However, the relationship between DT and the concentration of limonene in hemp EO was described by the exponential decay regression model and the fitted models are the same ($\hat{Y}=\text{7}.\text{5}\text{6}e^{-\text{0}.\text{0}\text{0}\text{8}DT}$) (Fig 2F). This compound showed a general decline in concentration with increasing DT; limonene concentration in the EO at 0–5 min DT was 8.3% and was reduced to 0.90% in the 240–280 min DT (Table 1). This pattern is further supported by hydrodistillation extraction kinetics regression models for *Juniperus virginiana*, where the highest concentration of limonene (43.0%) was observed in the 0–5 min fraction, compared to a significantly lower concentration (26.9%) in the continuous 0–240 min distillation control [52].

***β*-(E)-Ocimene** is a very common plant volatile compound, an important floral-scent compound, because of sweet herbal odor responsible for attracting floral visitors [69,70]. It has been found in a variety of plants, and it is also a significant compound of the hemp EO [67]. The concentration of these compounds in the control EO (0–180 min) was 5.46%. As observed with other monoterpenes, β-(E)-ocimene also has an Exponential decay regression model ($\hat{Y}=\text{7}.\text{8}\text{8}Exp(-\text{0}.\text{0}\text{0}\text{6}DT)$) (Fig 2G), and this compound also showed a general decline in concentration with increasing DT. Summarily, concentration in the EO at 0–5 min DT was 7.56% and was reduced to 1.83% in the 240–280 min DT (Table 1). A similar trend was observed during the distillation of lavender EO, where the concentration of *β*-ocimene progressively declined over the distillation period from 5 to 60 minutes [71].

***α*-Terpineol** has an odor similar to lilacs, and it is a common ingredient in perfumes, cosmetics, and other aroma industries [72]. It is also found in hemp [73]. *α*-Terpineol possess antioxidant, anti-inflammatory, anti-tumor, analgesic and anticonvulsant effects, and therefore, it has potential as pharmaceuticals as well [74,75]. In the control (0–180 min), this compound was presented with 2.67% in EO. However, the concentration of *α*-terpineol increased from 2.7% at 0–5 min DT to the max of 4.2% in the 40–80 min DT and then decreased again down to 1.4% in the 160–200 min DT EO (Table 1). This decreasing trend could be consequence of degradation of *α*-terpineol in water during distillation process [76].

If EO with high concentrations of monoterpene hydrocarbons such as *α*-pinene, *β*-pinene, myrcene, limonene, *β*-(E)-ocimene is desirable for specific applications, then such EO can be obtained when this type of high cannabinoids-hemp is distilled for 5- or 10-min. Hemp EO with a higher concentration of *α*-terpineol (oxygenated monoterpene) can be obtained when the EO fraction 40–80 min DT is collected separately from the other fractions. This is of exceptional commercial importance, because the targeted production of hemp EO with a higher proportion of monoterpenes can be significantly shortened, which would achieve great savings in DT and costs.

Hemp, EO rich in monoterpenes, possesses a unique and pleasant herbal aroma characterized by turpentine, woody, citrus, and floral notes. Its highly volatile top notes make it well suited for applications in the flavor and fragrance industries, as well as in aromatherapy, due to the documented benefits of individual monoterpenes [62,77,78]. Several studies have also confirmed that shorter DT effectively yield hemp EOs with higher monoterpene content—an attribute that enhances their value for aromatic and therapeutic applications [22,25].

### 3.3. Concentration of sesquiterpenes in hemp EO depends on distillation time (DT)

**Sesquiterpenes** (C_15_ compounds; three isoprene units) are bulkier than monoterpenes, and less volatile. Because of that, they were eluted late in the distillation process. The concentration of sesquiterpenes in the 0–5 min DT EO was 25.73%, then gradually increased to reach a plateau at 160–280 min DT (75.3–76.8%), while their concentration in the 0–180 min DT was 54.4% (Table 2). The relationship between DT and the concentration of sesquiterpenes (%) was best described by asymptotic (concave) regression model ($\hat{Y}=\text{7}\text{9}.\text{7}\text{3}-\text{5}\text{4}.\text{0}\text{6}e^{-\text{0}.\text{0}\text{1}\text{0}\text{5}DT}$) (Fig 2B). There were 18 sesquiterpenes in hemp EO: *β*-caryophyllene, *α*-humulene, *δ*-selinene, *γ*-cadinene, *δ*-cadinene, nerolidol, maaliol, germacrene D-4-ol, spathulenol, caryophyllene oxide, globulol, ledol, *γ*-eudesmol, caryophylla-4(12),8(13)-dien-5*α*-ol, *β*-eudesmol, *α*-eudesmol, bulnesol, and *α*-bisabolol (Table 2).

**Table 2:** Mean concentrations of sum sesquiterpenes and single sesquiterpenes: β-caryophyllene, α-humulene, δ-selinene, γ-cadinene, δ-cadinene, nerolidol, maaliol, germacrene D-4-ol, spathulenol, caryophyllene oxide, globulol, ledol, γ-eudesmol, caryophylla-4(12),8(13)-dien-5α-ol, β-eudesmol, α-eudesmol, bulnesol, and α-bisabolol obtained from the 10 distillation time (DT) frames and control (0-180 min) of top hemp plants. $\sqrt{MSE}=$ Square root of Mean Squares Error that estimates the common standard deviation (σ). Within each row, means sharing the same letter are not significantly different.

RI calc	RI lit	DT (min)	0-5	5-10	10-20	20-40	40-80	80-120	120-160	160-200	200-240	240-280	0-180	$\sqrt{MSE}$
		**Sesquiterpenes**	25.73 g	29.29 g	39.58 f	48.67 e	53.83 d	61.37 c	68.82 b	75.94 a	75.29 a	76.83 a	54.40 d	
**1419**	**1417**	**β-Caryophyllene**	6.78 f	8.80 cd	8.88 cd	7.48 ef	8.04 de	9.13 c	10.70 b	10.51 b	10.99 b	12.37 a	7.17 ef	0.337
**1451**	**1452**	**α-Humulene**	2.13 e	3.12 d	3.14 d	3.42 bcd	4.07 abc	3.38 bcd	4.10 abc	4.11 abc	4.21 ab	4.74 a	3.26 cd	0.233
**1494**	**1492**	**δ-Selinene**	0.66 g	0.78 f	0.890 e	0.947 de	1.06 bc	1.016 cd	1.11 bc	0.86 ef	1.12 abc	1.12 ab	1.22 a	0.034
**1514**	**1513**	**γ-Cadinene**	1.25 d	2.08 b	2.17 ab	2.36 ab	2.27 ab	2.22 ab	2.48 a	1.53 cd	2.51 a	2.41 ab	1.65 c	0.098
**1523**	**1522**	**δ-Cadinene**	0.87 f	1.01 ef	1.09 e	1.23 d	1.24 d	1.24 cd	1.29 bcd	1.45 a	1.40 ab	1.31 bcd	1.37 abc	0.042
**1530**	**1531**	**Nerolidol**	0.71 e	0.82 de	0.94 cd	1.10 bc	1.12 bc	1.18 b	1.17 b	1.55 a	1.24 b	1.12 bc	1.45 a	0.039
**1565**	**1566**	**Maaliol**	2.62 e	3.22 de	3.59 cd	4.15 bc	4.20 bc	4.42 b	4.65 b	5.83 a	4.55 b	4.75 b	3.71 cd	0.169
**1572**	**1574**	**Germacrene D-4-ol**	2.20 f	2.95 e	3.21 de	3.52 cd	3.67 cd	3.80 bc	3.91 bc	5.61 a	4.25 b	4.08 bc	2.66 ef	0.145
**1576**	**1577**	**Spathulenol**	1.56 e	1.73 e	1.88 e	2.37 d	2.70 cd	3.37 b	3.24 b	3.85 a	3.57 ab	3.37 b	2.86 c	0.098
**1580**	**1582**	**Caryophyllene oxide**	0.31 h	0.58 g	0.79 f	1.18 e	1.48 cd	1.61 bc	1.75 ab	1.46 cd	1.89 a	1.79 ab	1.37 de	0.048
**1588**	**1590**	**Globulol**	0.26 gh	0.62 fg	0.99 f	1.73 e	2.50 d	2.93 d	3.73 c	4.72 a	4.16 bc	4.41 ab	0.11 h	0.125
**1600**	**1602**	**Ledol**	ND	ND	ND	ND	ND	ND	ND	0.18 c	0.29 b	0.12 d	2.58 a	
**1627**	**1630**	**γ-Eudesmol**	0.31 g	0.68 fg	1.10 f	1.93 e	2.77 d	3.61 c	4.59 b	5.70 a	5.35 a	5.20 ab	3.06 cd	0.167
**1640**	**1639**	**Caryophylla-4(12),8(13)-dien-5α-ol**	ND	ND	0.34 f	0.60 e	0.72 e	1.44 a	1.10 c	1.34 ab	1.34 ab	1.22 bc	0.86 d	
**1646**	**1649**	**β-Eudesmol**	ND	0.12 g	0.21 g	0.39 f	0.60 e	0.79 d	0.90 c	0.98 bc	1.12 a	1.05 ab	0.70 d	
**1650**	**1652**	**α-Eudesmol**	0.26 g	0.61 fg	1.04 f	1.90 e	2.88 d	4.00 c	4.70 b	6.13 a	5.87 a	6.29 a	3.37 cd	0.166
**1669**	**1670**	**Bulnesol**	0.01 e	0.02 e	0.07 de	0.26 d	0.65 c	1.17 b	2.62 a	3.11 a	3.01 a	2.70 a	1.15 b	0.225
**1687**	**1685**	**α-Bisabolol**	ND	ND	1.24 f	2.22 ef	3.03 e	4.44 d	5.79 c	6.93 ab	6.20 bc	7.28 a	4.14 d	

The sesquiterpene profile observed in this study aligns with previously reported data on the EOs of various hemp accessions [6,11,79]. Additionally, sesquiterpenes have been reported to be more abundant in early harvests [80], as well as in the female inflorescences and leaves of spontaneous hemp varieties, which are recognized as significant sources of sesquiterpenes and CBD-rich EOs [81].

**Fig 3:**
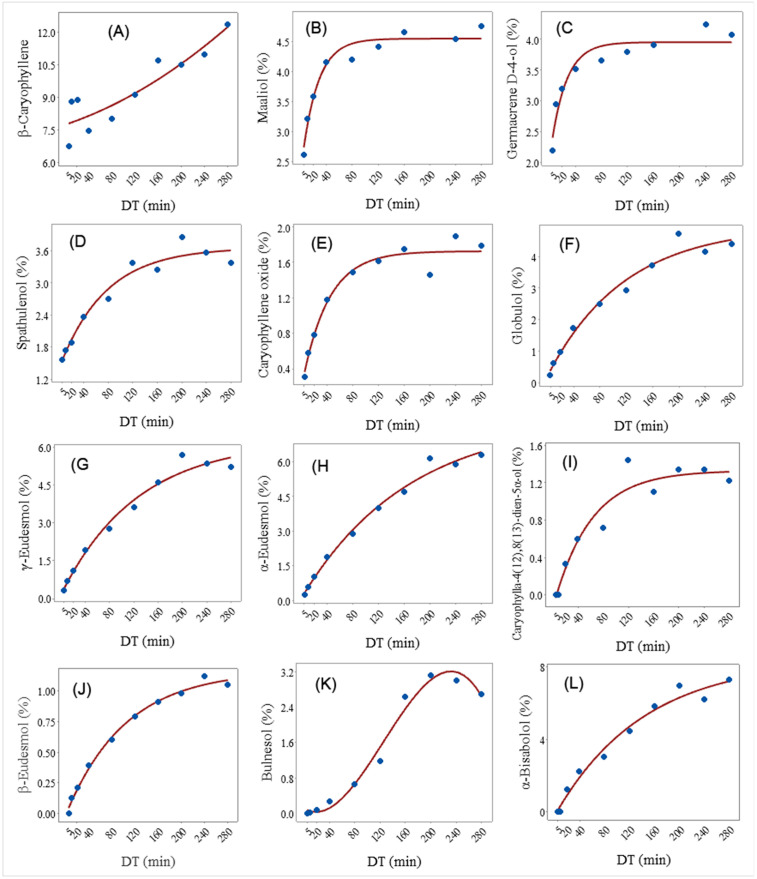
A fitted regression model that shows the relationship between distillation time (DT) and the concentration (%) of (A) β-caryophyllene, (B) maaliol, (C) germacrene D-4-ol, (D) spathulenol, (E) caryophyllene oxide, (F) globulol, (G) γ-eudesmol, (H) α-eudesmol, (I) caryophylla-4(12),8(13)-dien-5α-ol, (J) β-eudesmol, (K) bulnesol, and (L) α-bisabolol.

Sesquiterpenes are the most abundant class in hemp EO, with ***β*-caryophyllene** domination [8,46,82]. *β*-Caryophyllene possesses a spicy odor type, with sweet, woody, and clove undernotes, which gives a unique aroma to hemp EO. Additionally, promising biological activities such as antioxidant, anti-inflammatory, and anticancer make it very attractive for science and industry [83]. The concentration of *β*-caryophyllene in the control was 7.17%, while the EO of the fractions increased significantly with time; from 6.78% in the 0–5 DT EO to 12.37% in the 240–280 min DT EO (Table 2). *β*-Caryophyllene has a second order polynomial regression model (Adjusted R^2^ = 83.3%; $\hat{Y}=\text{7}.\text{7}\text{7}+\text{0}.\text{0}\text{0}\text{9}DT+\text{0}.\text{0}\text{0}\text{0}\text{0}\text{3}{DT}^{\text{2}}$) (Fig 3A). The results highlight *β*-caryophyllene as a functional non-psychoactive ligand in food, i.e., a dietary cannabinoid with anti-inflammatory properties [84]. Therefore, there is interest in discovering pretreatments that can increase its content in hemp EO, such as microwave or oven heating [32].

*β*-caryophyllene is usually found together with small quantities of its isomers among them *α*-humulene (formerly *α*-caryophyllene) and its oxidation product such as caryophyllene oxide and caryophylla-4(12),8(13)-dien-5*α*-ol [84]. ***α*-Humulene** has a woody odor, and it is identified as a major component in the hops (*Humulus lupulus* L.) EO, also a species that belongs to the Cannabaceae family [85]. Exploring the relationship between DT and the concentration of *α*-humulene (%) showed that it is best described by an asymptotic (concave) regression model ($\hat{Y}=\text{4}.\text{3}\text{9}-\text{1}.\text{8}\text{3}e^{-\text{0}.\text{0}\text{1}\text{2}\text{6}DT}$) (Fig 2H). *α*-Humulene concentration in the 0–5 min DT was 2.13% and increased to 4.74% in the 240–280 min DT, while its concentration in the control EO was 3.26% (Table 2).

Compounds such as *δ*-selinene and *γ*-cadinene are not for fragrance use. However, they are known as surfactants and emulsifiers [86]. The relationship between DT and the concentration of ***δ*-selinene** was best described by asymptotic (concave) regression model ($\hat{Y}=\text{1}.\text{0}\text{9}\text{3}-\text{0}.\text{4}\text{8}e^{-\text{0}.\text{0}\text{3}\text{5}DT}$) (Fig 2I) The concentration of *δ*-selinene, as well as sesquiterpenes increase generally with DT, from 0.66% in the 0–5 min DT to 1.12% in the 240–280% DT. On the other side, its concentration in the control DT was 1.22% (Table 2).

The concentration of ***γ*-cadinene** was the lowest in the 0–5 min DT EO (1.25%), then it increases and maintains a plateau from 10–20–120–160 min (2.48%), then there was a slight dip at 160–200 min and a recovery in the 200–240 min (1.51%) (Table 2). The fluctuations observed in *γ*-cadinene concentration in different DT ($\hat{Y}=\text{2}.\text{3}\text{5}-\text{3}.\text{9}\text{6}e^{-\text{0}.\text{2}\text{5}\text{6}\text{3}DT}$) (Fig 2J), especially the dip at 160–200 min, suggests that the dynamics of *γ*-cadinene release were influenced by multiple factors throughout the distillation process.

However, ***δ*-cadinene** is sesquiterpene with mild herbal and woody odor [87], responsible for the antimicrobial and acaricidal activity [88,89]. The relationship between DT and the concentration of *δ*-cadinene (%) was described well by asymptotic (concave) regression model ($\hat{Y}=\text{1}.\text{3}\text{3}\text{8}-\text{0}.\text{4}\text{9}\text{7}e^{-\text{0}.\text{0}\text{3}\text{1}DT}$) (Fig 2K). There was a gradual increase in the concentration of *δ*-cadinene from the initial 0–5 min (0.87%) until around 160–200 min (1.45%); *δ*-Cadinene in the 200–240 min DT EO was not significantly different (Table 2). However, in the control sample, content of *δ*-cadinene was 1.37%.

Oxygenated sesquiterpene **nerolidol** is a major contributor of the characteristic floral aroma note [90]. In the control sample (0–180 min), its concentration was 1.45%, and it was not statistically different from the peak value, although slightly lower numerically. The concentration of nerolidol increases steadily over the DT, starting from 0.714% at 0–5 min and reaching a peak at 160–200 min (1.546%) (Table 2). This suggests that nerolidol is continuously released or formed during the distillation process. After reaching its peak, there was a decrease in nerolidol concentration at 200–240 min (1.24%) and a further decline at 240–280 min (1.121%). This relation can be described by asymptotic (concave) regression model ($\hat{Y}=\text{1}.\text{1}\text{7}-\text{0}.\text{5}\text{8}e^{-\text{0}.\text{0}\text{4}\text{8}DT}$) (Fig 2L).

Oxygenated sesquiterpene **maaliol**, found in patchouli oil (*Pogostemon cablin*), *Valeriana wallichii* and several other plants, possesses cytotoxic and insecticidal activity [91,92]. The connection between DT and the concentration of maaliol (%) was best described by asymptotic (concave) regression model models ($\hat{Y}=\text{4}.\text{5}\text{5}-\text{2}.\text{2}\text{2}\text{4}e^{-\text{0}.\text{0}\text{4}\text{1}DT}$) (Fig 3B). The trend was similar to the one with *γ*-cadinene. There was a noticeable and consistent increase in the concentration of maaliol in the EO fraction from the beginning of the distillation process (2.62%) up to 160–200 min (5.83%). After that, there was a slight decrease in the concentration of maaliol in the next two DT (Table 2). The concentration of this compound in the 0–180 min control was 3.71%.

Sesquiterpenoid **germacrene D-4-ol** showed insecticidal activity [93]. The relationship between the concentration of sesquiterpene compounds **germacrene D-4-ol** and DT in the hemp EO was best described by asymptotic (concave) regression model ($\hat{Y}=\text{3}.\text{9}\text{6}-\text{1}.\text{9}\text{2}e^{-\text{0}.\text{0}\text{4}\text{3}DT}$) (Fig 3C). There was a general trend of increasing concentration of Germacrene D-4-ol in the EO with the extension of DT, from 2.20% at 0–5 min DT EO to 5.61% in the 160–200 min EO (Table 3). After that, germacrene D-4-ol decreased in the EO of 200–240 min and in 240–280 min DT EO. Germacrene D-4-ol concentration in the 0–180 control was 2.66% (Table 2).

**Table 3:** Mean concentrations of sum cannabinoids, CBD, Cannabichromene, δ8-Tetrahydrocannabinol, δ9-Tetrahydrocannabinol, and other EO constituents (acid asters, ketone, alcohol) obtained from the 10 distillation time (DT) frames and control (0-180 min) of top hemp plants. $\sqrt{MSE}$= Square root of the Mean Squares Error that estimates the common standard deviation (σ). Within each row, means sharing the same letter are not significantly different.

RI calc	RI lit	DT (min)	0-5	5-10	10-20	20-40	40-80	80-120	120-160	160-200	200-240	240-280	0-180	$\sqrt{MSE}$
		**Cannabinoids**	ND	ND	0.21 h	0.60 h	1.25 g	2.58 f	3.40 e	4.29 d	5.53 c	7.12 b	8.37 a	
**1419**	**1417**	**CBD**	ND	ND	0.21 gh	0.60 g	1.25 f	2.51 e	3.32 d	4.15 c	5.35 b	7.12 a	7.64 a	0.152
**1451**	**1452**	**Cannabichromene**	ND	ND	ND	ND	ND	0.07 d	0.08 d	0.14 c	0.17 b	ND	0.37 a	
**1494**	**1492**	**δ8-Tetrahydrocannabinol**	ND	ND	ND	ND	ND	ND	ND	ND	ND	ND	0.10	
**1514**	**1513**	**δ9-Tetrahydrocannabinol (Dronabinol)**	ND	ND	ND	ND	ND	ND	ND	ND	ND	ND	0.17	
		**Others EO constituents (acid asters, ketone, alcohol)**	2.712 a	2.461 ab	2.19 abc	2.36 ab	1.76 cd	1.22 de	0.90 e	0.79 e	0.91 e	0.73 e	2.04 bc	

**Spathulenol** is sesquiterpene with numerous biological activities: anticholinesterase, antinociceptive, anti-hyperalgesic, anti-mycobacterial, antioxidant, anti-proliferative, anti-oedematogenic, and anticancer [94]. The relationship between spathulenol (%) in the EO and DT was best described by asymptotic (concave) regression model ($\hat{Y}=\text{3}.\text{6}\text{5}\text{6}-\text{2}.\text{2}\text{5}e^{-\text{0}.\text{0}\text{1}\text{3}DT}$) (Fig 3D). The concentration of spathulenol in the DT fractions followed similar trend to the one of germacrene-D-4ol, with low concentration in the first 3 DT EO and gradually increasing to 3.85% in the 160–200 min DT EO, and then decreased slightly in the subsequent DT EO (Table 2). Spathulenol concentration in the 0–180 control EO was 2.86%.

**Caryophyllene oxide** has a woody odor with a sweet, dry, fresh and spicy scent. It possesses numerous biological activities, among them anti-inflammatory, analgesic, gastroprotective, and anticancer [95]. The relationship between caryophyllene oxide and DT was best described by asymptotic (concave) regression model ($\hat{Y}=\text{1}.\text{7}\text{2}\text{3}-\text{1}.\text{5}\text{3}\text{7}e^{-\text{0}.\text{0}\text{2}\text{5}DT}$) (Fig 3E). Caryophyllene oxide concentration was low in the initial DT 0–5 min (0.313%) and increased gradually to max out at 200–240 min DT (1.893%). Its concentration in the 0–180 min control EO was 1.365% (Table 2).

**Globulol** is sesquiterpene alcohol with strong antifungal activity [96]. The asymptotic (concave) regression model ($\hat{Y}=\text{4}.\text{9}\text{7}-\text{4}.\text{7}\text{9}e^{-\text{0}.\text{0}\text{0}\text{8}\text{6}DT}$) described best the relationship between globulol concentrations in the EO and the DT (Fig 3F). The concentration of this compound in the 0–5 min DT EO was 0.26% and gradually increased with the increase in DT to reach 4.72% in the 160–200 min DT EO (Table 2). Interestingly, the concentration of globulol in the 0–180 min DT EO was only 0.111, suggesting possible loss or conversion of this compound into another one (Table 2). Cavalcanti et al. [97] reported that globulol was eluted after the second hour of distillation of *Schinus terebinthifolius* and *Schinus mole*. Furthermore, in a study on six medical *Cannabis sativa* genotypes, two of the genotypes did not contain globulol [98].

**Ledol** is poisonous sesquiterpene which affects the central nervous system [99]. The concentration of ledol in the DT EO from 0 until 160 min DT was zero, suggesting that this compound starts eluting late in the distillation process, reading a maximum of 0.29% in the 200–240 min DT EO (Table 2). These results are similar to those in a previous report on distillation study of *Schinus terebinthifolius* and *Schinus mole* using hydrodistillation; ledol was extracted late in the process, after 2 h of hydrodistillation [97].

**γ-Eudesmol** is oxygenized sesquiterpene with a waxy sweet odor and multiple biological activities [100]. The relationship between the DT and the concentration of γ-eudesmol (%) was best described by asymptotic (concave) regression model ($\hat{Y}=\text{6}.\text{2}\text{4}-\text{6}.\text{0}\text{9}e^{-\text{0}.\text{0}\text{0}\text{8}DT}$) (Fig 3G). The trend was similar to that of globulol; the concentration of γ-eudesmol was low (0.31%) in the 0–5 DT EO and increased gradually to reach max at 160–200 min DT EO (5.67%) (Table 2). The concentration of this compound did not change in the subsequent two DT fractions. The concentration of γ-eudesmol in the control 0–180 EO was 3.058%.

**Caryophylla-4(12),8(13)-dien-5*α*-ol** is formed by oxidation of *β*-caryophyllene [101], and in small concentrations it can be found in *Baccharis* species [102], as well as *Achillea* sp. [103] and others. The relationship between DT and the concentration of caryophylla-4(12),8(13)-dien-5*α*-ol in hemp EO was best described by asymptotic (concave) regression model ($\hat{Y}=\text{1}.\text{3}\text{3}-\text{1}.\text{4}\text{8}e^{-\text{0}.\text{0}\text{1}\text{7}DT}$) (Fig 3I). The compound did not elute until 10–20 min (0.336%), increased gradually to reach max at 80–120 min DT (1.443%), while its concentration in the 0–180 min control EO was 0.864% (Table 2).

***β*-Eudesmol** is sesquiterpenoid alcohol and is isolated from various plants, and it is very potent anticancer compound [104]. The concentration of β-eudesmol (%) vs DT was best described by the asymptotic (concave) regression model ($\hat{Y}=\text{1}.\text{1}\text{6}-\text{1}.\text{1}\text{7}e^{-\text{0}.\text{0}\text{1}DT}$) (Fig 3J). *β*-Eudesmol was absent from the 0–5 min DT EO but did appear in the 5–10 min DT EO (0.124%), then increased gradually to reach maximum at 200–240 min DT EO (1.115%). The concentration of this compound in the 0–180 min DT was 0.696%, similar to the one in the 80–120 min DT EO (Table 2).

***α*-Eudesmol**, sesquiterpenoid alcohol have a potential for treating neurogenic inflammation in the trigemino-vascular system such as migraine [105]. The relationship between DT and the concentration of α-eudesmol (%) was best described by the asymptotic (concave) regression model ($\hat{Y}=\text{7}.\text{8}\text{7}-\text{7}.\text{7}\text{5}e^{-\text{0}.\text{0}\text{0}\text{6}DT}$) (Fig 3H). The concentration of *α*-eudesmol was low in the 0–5 min DT EO (0.255%) and increased gradually to reach a maximum in the later DT, 160–280 min DT fractions (5.8–6.2%). The concentration of this compound in the 0–180 min DT control was 3.373%, similar to the compound concentration in the 40–80 and 80–120 min DT (Table 2).

**Bulnesol** is spicy odor sesquiterpene alcohol present in large amounts in *Bulnesia sarmientoi* [106], but it can also be found in other plants in minor concentrations. The relationship between DT and the concentration of bulnesol (%) was best described by polynomial regression model (adjusted R^2^ = 98.5%; $\hat{Y}=\text{0}.\text{1}\text{1}-\text{0}.\text{0}\text{0}\text{8}\text{8}DT+\text{0}.\text{0}\text{0}\text{0}\text{2}\text{5}DT^{\text{2}}$) (Fig 3K). The concentration of bulnesol was very low in the 0–5 min DT EO (0.003%), but then it increased gradually to reach a plateau at 120–280 min (2.6–3.1%). The concentration of this compound in the control EO was 1.152% (Table 2).

***α*-Bisabolol** is a monocyclic sesquiterpene alcohol with floral odor type (with nascence of peppery, balsamic and clean notes), derived from a variety of plants, present in higher concentrations in chamomile EO. It is widely used in cosmetics and pharmacies because of its anti-inflammatory, anticancer, and other activities [107,108]. The relationship between DT and the concentration of *α*-bisabolol (%) was best described by asymptotic (concave) regression model ($\hat{Y}=\text{8}.\text{3}\text{7}-\text{8}.\text{6}\text{3}e^{-\text{0}.\text{0}\text{0}\text{7}DT}$) (Fig 3L). α-Bisabolol was not detected in the initial DT of 0–5 and 5–10 min, then it eluted and accumulated in the 10–20 min (1.242%) (Table 2). After that, the concentration of this compound gradually increased to reach a maximum later in the DT, at 240–280 min (7.284%). The concentration of *α*-bisabolol in the control EO was 4.141% and not significantly different from the one in the 80–120 min DT EO fraction (Table 2).

Unlike monoterpenes, a higher accumulation of sesquiterpenes was recorded in the period after 40 min. This indicates the fact that for a higher concentration of sesquiterpenes in the EO, a longer distillation is necessary. The concentration of some sesquiterpenes increases even after 180 min, which is the standard distillation time of hemp EO (control). Greater share of sesquiterpenes in the EO influences the fragrance profile of hemp EO as it gets a more pronounced spicy note, with earthy and woody odors, similar as in hop EO [109]. Prolonged distillation time gives EO with a different chemical profile, but also increases costs, so it is recommended that the duration of the process be adjusted to the potential application. Practically, the most important sesquiterpenes in hemp EO are *β*-caryophyllene and *α*-humulene, due to their abundance and biological significance. Other sesquiterpenes, which are primarily bi-products of *β*-caryophyllene degradation, are considered to have minor significance in hemp EO quality and biological activity [9,32,110]. In brief, prolonging the distillation time can lead to an increase in sesquiterpenes, which are heavier and less volatile compounds [111], a finding that has also been confirmed by our research.

### 3.4. Concentration of cannabinoids in hemp EO depends on distillation time (DT)

**Fig 4:**
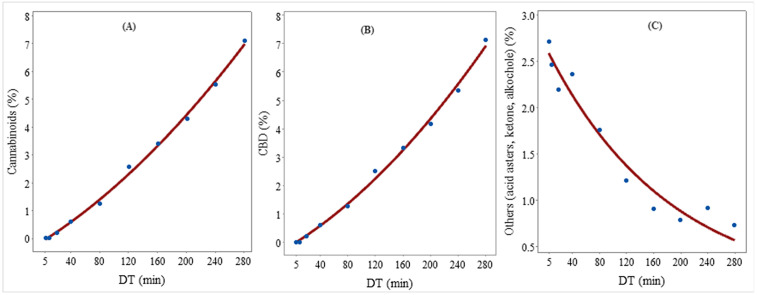
Fitted regression model that shows the relationship between distillation time (DT) and the concentration (%) of (A) Cannabinoids [$\hat{Y}=-\text{0}.\text{1}\text{2}\text{4}+\text{0}.\text{0}\text{1}\text{7}DT+\text{0}.\text{0}\text{0}\text{0}\text{0}\text{3}{DT}^{\text{2}}$], (B) CBD [$\hat{Y}=-\text{0}.\text{0}\text{9}\text{4}+\text{0}.\text{0}\text{1}\text{5}DT+\text{0}.\text{0}\text{0}\text{0}\text{0}\text{4}{DT}^{\text{2}}$], and (C) Others (acid asters, ketone, alkochole) [$\hat{Y}=\text{2}.\text{6}\text{6}e^{\left(-\text{0}.\text{0}\text{0}\text{5}\text{5}DT\right)}$].).

Cannabinoids belong to meroterpenoids made of 21 or 22 atoms and a pentyl or propyl side chain (i.e., prenylated polyketides) [112]. The relationship between DT and the concentration of total cannabinoids (%) can be described well by a second order polynomial regression model (Adjusted R^2^ = 99.2%; $\hat{Y}=-\text{0}.\text{1}\text{2}\text{4}+\text{0}.\text{0}\text{1}\text{6}\text{5}DT+\text{0}.\text{0}\text{0}\text{0}\text{0}\text{3}DT^{\text{2}}$) (Fig 4A). The concentration of the total cannabinoids was below the detection limit in the 0–10 min DT fraction, then increased gradually to reach 7.1% in the 240–280 EO fraction, while their concentration in the non-stop 0–180 min EO was 8.27% (Table 3). Indeed, this trend was similar to the one observed with the CBD concentration in the EO fractions, indicating the total cannabinoids were mostly CBD. A previous study found that the duration of distillation significantly affected the chemical composition of EOs, leading to variations in terpene extraction while generally increasing the presence of minor sesquiterpenes and CBD [25], which is in agreement with the results of this study. Additionally, cannabinoids had a strong positive correlation with sesquiterpenes [79].

**Cannabidiol (CBD):** The relationship between DT and the concentration of CBD (%) was best described by a second order polynomial regression model (Adjusted R^2^ = 99.5%; $\hat{Y}=-\text{0}.\text{0}\text{9}\text{4}+\text{0}.\text{0}\text{1}\text{5}\text{2}DT+\text{0}.\text{0}\text{0}\text{0}\text{0}\text{3}\text{5}DT^{\text{2}}$) (Fig 4B). Similar to the trend in *α*-bisabolol, the concentration of CBD in the 0–5 and in the 5–10 min DT fractions was below the detection limit (0.00%). Cannabidiol appeared in the EO in the 10–20 min DT and then gradually increased to reach a maximum of 7.124% in the 240–280 min DT (Table 3). Interestingly, the concentration of CBD in the control EO (0–180 min) of 7.636% was similar to the one in the 240–280 min DT EO (Table 3). This may suggest conversion of some compounds into CBD in the non-stop distillation of 0–180 min.

**Cannabichromene (CBC)** was eluted late in the distillation process; its concentration in the EO fractions up to 80 min was below the detection limit (Table 3). The compound appeared in the 80–120 min DT EO (0.071%), and gradually increased to 0.173% in the 200–240 min DT EO. Interestingly, CBC was not detected in the 240–280 min DT EO. Also, the CBC concentration in the non-stop 0–180 min EO was 0.367% (Table 3).

**δ8-Tetrahydrocannabinol:** The concentration of δ8-Tetrahydrocannabinol in all EO fractions was below the detection limit, while its concentration in the non-stop 0–180 min control was 0.096% (Table 3).

**δ9-Tetrahydrocannabinol (Dronabinol):** The concentration of δ9-Tetrahydrocannabinol (Dronabinol) in all EO fractions was below the detection limit, and its concentration in the 0–180 min non-stop distillation EO was 0.166% (Table 3).

The DT for extracting cannabinoids from hemp can vary, but a common range is between 180 and 240 min [113]. However, the optimal DT can depend on factors like the specific cultivar, the desired composition of the EO, and the extraction method used. Some studies have found that longer DT can lead to increased levels of certain cannabinoids due to decarboxylation reactions [33,114,115].

### 3.5. Concentration of other compounds in hemp EO depend on distillation time (DT)

The relationship between DT and the concentration of others EO constituents **(acid asters, ketone, alkochol**) (%) is described by asymptotic regression model ($\hat{Y}=\text{2}.\text{6}\text{6}e^{-\text{0}.\text{0}\text{0}\text{5}\text{5}DT}$) (Fig 4C). The concentration of these constituents in the EO generally decreased with an increase in DT and varied between 2.7% (in the 0–5 min EO) to 0.734% (Table 3). The concentration of these constituents in the 0–180 min DT was 2.04%. Therefore, if desired, an EO fraction with a relatively high concentration of these constituents can be obtained by distilling the biomass for a shorter duration.

## 4. Conclusions

This study confirmed that EO constituents from high-cannabinoid *C. sativa* elute at distinct times during hydrodistillation, enabling the production of chemically diverse EO fractions. By analyzing ten sequential distillation timeframes, research showed clear temporal patterns: monoterpenes appeared early, while cannabinoids eluted later. Regression analysis supported these trends, demonstrating that distillation timing can be used to selectively enrich or exclude specific compounds. This time-based fractionation offers a practical strategy for customizing EO compositions to suit various applications, such as aromatherapy, cosmetics, or cannabinoid-rich wellness products, and aids in regulatory compliance. The approach also enhances distillation efficiency and product consistency. Future research should explore the bioactivity, sensory properties, and functional uses of these EO fractions, as well as test the method across different hemp varieties and processing conditions.

## Supporting information

S1 File Supporting Information Zheljazkov et al. Data_r2. (XLSX)

## References

[1] Brar KK, Raheja Y, Chadha BS, Magdouli S, Brar SK, Yang YH, et al. A paradigm shift towards production of sustainable bioenergy and advanced products from *Cannabis*/hemp biomass in Canada. Biomass Conv Bioref. 2024;14:3161–82. doi: 10.1007/s13399-022-02570-6 PMC8934023 35342682

[2] Muedi HTH, Kujoana TC, Shai K, Mabelebele M, Sebola NA. The use of industrial hemp (Cannabis sativa) on farm animal’s productivity, health and reproductive performance: a review. Anim Prod Sci. 2024;64(2). doi: 10.1071/an23268

[3] Tănase Apetroaei V, Pricop EM, Istrati DI, Vizireanu C. Hemp Seeds (Cannabis sativa L.) as a Valuable Source of Natural Ingredients for Functional Foods-A Review. Molecules. 2024;29(9):2097. doi: 10.3390/molecules29092097 38731588 PMC11085560

[4] Aguzzi C, Perinelli DR, Cespi M, Zeppa L, Mazzara E, Maggi F, et al. Encapsulation of hemp (*Cannabis sativa* L.) essential oils into nanoemulsions for potential therapeutic applications: Assessment of cytotoxicological profiles. Molecules. 2023;28: 6479. doi: 10.3390/molecules28186479 37764255 PMC10537312

[5] Mazzara E, Spinozzi E, Maggi F, Petrelli R, Fiorini D, Scortichini S, et al. Hemp (Cannabis sativa cv. Kompolti) essential oil and its nanoemulsion: Prospects for insecticide development and impact on non-target microcrustaceans. Industrial Crops and Products. 2023;203:117161. doi: 10.1016/j.indcrop.2023.117161

[6] Di Sotto A, Gullì M, Acquaviva A, Tacchini M, Di Simone SC, Chiavaroli A, et al. Phytochemical and pharmacological profiles of the essential oil from the inflorescences of the Cannabis sativa L. Industrial Crops and Products. 2022;183:114980. doi: 10.1016/j.indcrop.2022.114980

[7] Marques SdPPM, Pinheiro RO, Nascimento RAd, Andrade EHdA, Faria LJGd. Effects of harvest time and hydrodistillation time on yield, composition, and antioxidant activity of mint essential oil. Molecules. 2023;28:7583. doi: 10.3390/molecules28227583 38005307 PMC10675317

[8] Šovljanski O, Aćimović M, Sikora V, Koren A, Saveljić A, Tomić A, et al. Exploring (Un)Covered Potentials of Industrial Hemp (Cannabis sativa L.) Essential Oil and Hydrolate: From Chemical Characterization to Biological Activities. Natural Product Communications. 2024;19(7). doi: 10.1177/1934578x241264712

[9] Kabdy H, Abdelmounaim B, Aitbaba A, Hajar A, Yasmine J, Oufquir S, et al. Moroccan Cannabis sativa essential oil attenuates peripheral neuropathic pain induced by chronic sciatic nerve constriction injury in mice. J Ethnopharmacol. 2025;343:119486. doi: 10.1016/j.jep.2025.119486 39947371

[10] Singh MK, Sarita S, Singh S, Mishra S, Shankar U, Maurya A, et al. Investigating the phytochemical diversity and anti-inflammatory activity of a non-psychoactive genotype of Cannabis sativa L. from India. Microchemical Journal. 2025;211:113111. doi: 10.1016/j.microc.2025.113111

[11] Abdollahi M, Sefidkon F, Calagari M, Mousavi A, Mahomoodally MF. Impact of four hemp (Cannabis sativa L.) varieties and stage of plant growth on yield and composition of essential oils. Industrial Crops and Products. 2020;155:112793. doi: 10.1016/j.indcrop.2020.112793

[12] Pieracci Y, Fulvio F, Isca V, Pistelli L, Bassolino L, Montanari M, et al. The phenological stage of hemp inflorescences affects essential oil yield and its chemical composition. Industrial Crops and Products. 2023;197:116605. doi: 10.1016/j.indcrop.2023.116605

[13] André A, Leupin M, Kneubühl M, Pedan V, Chetschik I. Evolution of the polyphenol and terpene content, antioxidant activity and plant morphology of eight different fiber-type cultivars of *Cannabis sativa* L. cultivated at three sowing densities. Plants. 2020;9:1740. doi: 10.3390/plants9121740 33317167 PMC7763896

[14] Ascrizzi R, Ceccarini L, Tavarini S, Flamini G, Angelini LG. Valorisation of hemp inflorescence after seed harvest: Cultivation site and harvest time influence agronomic characteristics and essential oil yield and composition. Industrial Crops and Products. 2019;139:111541. doi: 10.1016/j.indcrop.2019.111541

[15] Campiglia E, Radicetti E, Mancinelli R. Plant density and nitrogen fertilization affect agronomic performance of industrial hemp ( Cannabis sativa L.) in Mediterranean environment. Industrial Crops and Products. 2017;100:246–54. doi: 10.1016/j.indcrop.2017.02.022

[16] Meier C, Mediavilla V. Factors influencing the yield and the quality of hemp essential oil. J Int Hemp Assoc. 1998;5:16–20.

[17] Vuerich M, Ferfuia C, Zuliani F, Piani B, Sepulcri A, Baldini M. Yield and Quality of Essential Oils in Hemp Varieties in Different Environments. Agronomy. 2019;9(7):356. doi: 10.3390/agronomy9070356

[18] Kwaśnica A, Pachura N, Carbonell-Barrachina ÁA, Issa-Issa H, Szumny D, Figiel A, et al. Effect of Drying Methods on Chemical and Sensory Properties of Cannabis sativa Leaves. Molecules. 2023;28(24):8089. doi: 10.3390/molecules28248089 38138578 PMC10745367

[19] Wanas AS, Radwan MM, Chandra S, Lata H, Mehmedic Z, Ali A, et al. Chemical Composition of Volatile Oils of Fresh and Air-Dried Buds of Cannabis chemovars, Their Insecticidal and Repellent Activities. Natural Product Communications. 2020;15(5). doi: 10.1177/1934578x20926729

[20] Strzelczyk M, Chudy M, Łochyńska M, Gimbut M, Krawczyk K. Influence of Cultivar, Harvest Date, and Selected Weather Conditions on the Essential Oils Content in Inflorescences of Hemp Cannabis sativa L. Journal of Natural Fibers. 2023;20(1). doi: 10.1080/15440478.2022.2163332

[21] Mazzara E, Carletti R, Petrelli R, Mustafa AM, Caprioli G, Fiorini D, et al. Green extraction of hemp (*Cannabis sativa* L.) using microwave method for recovery of three valuable fractions (essential oil, phenolic compounds and cannabinoids): a central composite design optimization study. J Sci Food Agric. 2022;102:6220–6235. doi: 10.1002/jsfa.11971 35485728 PMC9790304

[22] Micalizzi G, Alibrando F, Vento F, Trovato E, Zoccali M, Guarnaccia P, et al. Development of a novel microwave distillation technique for the isolation of *Cannabis sativa* L. essential oil and gas chromatography analyses for the comprehensive characterization of terpenes and terpenoids, including their enantio-distribution. Molecules. 2021;26:1588. doi: 10.3390/molecules26061588 33805665 PMC8000122

[23] Pino S, Espinoza L, Jara-Gutiérrez C, Villena J, Olea AF, Díaz K. Study of Cannabis Oils Obtained from Three Varieties of C. sativa and by Two Different Extraction Methods: Phytochemical Characterization and Biological Activities. Plants (Basel). 2023;12(9):1772. doi: 10.3390/plants12091772 37176831 PMC10180737

[24] Lazarjani MP, Young O, Kebede L, Seyfoddin A. Processing and extraction methods of medicinal cannabis: a narrative review. J Cannabis Res. 2021;3:32. doi: 10.1186/s42238-021-00087-9 34281626 PMC8290527

[25] Palmieri S, Maggio F, Pellegrini M, Ricci A, Serio A, Paparella A, et al. Effect of the Distillation Time on the Chemical Composition, Antioxidant Potential and Antimicrobial Activity of Essential Oils from Different Cannabis sativa L. Cultivars. Molecules. 2021;26(16):4770. doi: 10.3390/molecules26164770 34443356 PMC8399774

[26] Zheljazkov VD, Sikora V, Semerdjieva IB, Kačániová M, Astatkie T, Dincheva I. Grinding and fractionation during distillation alter hemp essential oil profile and its antimicrobial activity. Molecules. 2020;25:3943. doi: 10.3390/molecules25173943 32872359 PMC7504750

[27] Benelli G, Pavela R, Petrelli R, Cappellacci L, Santini G, Fiorini D, et al. The essential oil from industrial hemp (Cannabis sativa L.) by-products as an effective tool for insect pest management in organic crops. Industrial Crops and Products. 2018;122:308–15. doi: 10.1016/j.indcrop.2018.05.032

[28] Chen C, Pan Z. Cannabidiol and terpenes from hemp – ingredients for future foods and processing technologies. Journal of Future Foods. 2021;1(2):113–27. doi: 10.1016/j.jfutfo.2022.01.001

[29] Novak J, Zitterl‐Eglseer K, Deans SG, Franz CM. Essential oils of different cultivars of Cannabis sativa L. and their antimicrobial activity. Flavour & Fragrance J. 2001;16(4):259–62. doi: 10.1002/ffj.993

[30] Orlando G, Adorisio S, Delfino D, Chiavaroli A, Brunetti L, Recinella L, et al. Comparative investigation of composition, antifungal, and anti-inflammatory effects of the essential oil from three industrial hemp varieties from Italian cultivation. Antibiotics. 2021;10:334. doi: 10.3390/antibiotics10030334 33809983 PMC8005080

[31] Bertoli A, Tozzi S, Pistelli L, Angelini LG. Fibre hemp inflorescences: From crop-residues to essential oil production. Industrial Crops and Products. 2010;32(3):329–37. doi: 10.1016/j.indcrop.2010.05.012

[32] Fiorini D, Molle A, Nabissi M, Santini G, Benelli G, Maggi F. Valorizing industrial hemp (Cannabis sativa L.) by-products: Cannabidiol enrichment in the inflorescence essential oil optimizing sample pre-treatment prior to distillation. Industrial Crops and Products. 2019;128:581–9. doi: 10.1016/j.indcrop.2018.10.045

[33] Zheljazkov VD, Maggi F. Valorization of CBD-hemp through distillation to provide essential oil and improved cannabinoids profile. Sci Rep. 2021;11:19890. doi: 10.1038/s41598-021-99335-4 34615971 PMC8494916

[34] Vozza Berardo ME, Mendieta JR, Villamonte MD, Colman SL, Nercessian D. Antifungal and antibacterial activities of *Cannabis sativa* L. resins. J Ethnopharmacol. 2024;318:116839. doi: 10.1016/j.jep.2023.116839 37400009

[35] Moreno-Chamba B, Salazar-Bermeo J, Hosseinian F, Martin-Bermudo F, Aguado M, De la Torre R, et al. Aromatic and cannabinoid profiles of Cannabis inflorescences and seed oils: A comprehensive approach for variety characterization. Industrial Crops and Products. 2024;210:118143. doi: 10.1016/j.indcrop.2024.118143

[36] European Union Plant Variety Database. Agricultural Species–Varieties. Hemp–*Cannabis sativa*. 2020. (accessed on 8 August 2024). Available online: https://ec.europa.eu/food/plant-variety-portal/

[37] Zheljazkov VD, Sikora V, Dincheva I, Kačániová M, Astatkie T, Semerdjieva IB, et al. Industrial, CBD, and wild hemp: How different are their essential oil profile and antimicrobial activity? Molecules. 2020;25:4631. doi: 10.3390/molecules25204631 33053634 PMC7587197

[38] SAS Institute Inc. SAS/STAT® 9.4 User’s Guide. Cary, NC: SAS Institute Inc.; 2016.

[39] Montgomery DC. Design and analysis of experiments, 10th Ed. Wiley, New York, USA: SAS Institute Inc.; 2016. SAS/STAT® 9.4 User’s Guide. SAS Institute Inc., Cary, NC; 2020. pp1–688.

[40] Bates DM, Watts DG. Nonlinear Regression and its Applications. New York: Wiley ; 2007. p. 1–365.

[41] Sumalini M, Pushpa TN, Srikantprasad D, Hiremath JS, Kerutagi MG, Mahantesha Naika BN, Ganga M. Study on influence of distillation duration on yield and composition of turmeric leaf essential oil. Eco Env Cons. 2024;30:657–660. doi: 10.53550/EEC.2024.v30i02.041

[42] Zheljazkov VD, Astatkie T, Jeliazkova EA, Schlegel V. Distillation time alters essential oil yield, composition, and antioxidant activity of male *Juniperus scopulorum* trees. J Oleo Sci. 2012;61:537–546. doi: 10.5650/jos.61.537 23018851

[43] Widayat W, Dita AS, Bambang C, Hantoro S. Study of Rendement of Red Ginger Essential Oil from Red Ginger Waste by Using Steam Distillation Process. E3S Web Conf. 2018;73:07002. doi: 10.1051/e3sconf/20187307002

[44] Petrović M, Petrović V, Mlinar Z, Babić S, Jukić J, Prebeg T, et al. Duration of Steam Distillation Affects Essential Oil Fractions in Immortelle (Helichrysum italicum). Horticulturae. 2024;10(2):183. doi: 10.3390/horticulturae10020183

[45] Juliano CCA, Mattu I, Marchetti M, Usai M. Chemical Characterization and Evaluation of the Antimicrobial Activity of Extracts from Two Cultivars of Cannabis sativa L. (Tisza and Kompolti) Grown in Sardinia. Applied Sciences. 2024;14(8):3353. doi: 10.3390/app14083353

[46] Luca SV, Wojtanowski K, Korona-Głowniak I, Skalicka-Woźniak K, Minceva M, Trifan A. Spent Material Extractives from Hemp Hydrodistillation as an Underexplored Source of Antimicrobial Cannabinoids. Antibiotics (Basel). 2024;13(6):485. doi: 10.3390/antibiotics13060485 38927152 PMC11201062

[47] El-Mernissi R, El Menyiy N, Moubachir R, Zouhri A, El-Mernissi Y, Siddique F, et al. Cannabis sativa L. essential oil: Chemical composition, anti-oxidant, anti-microbial properties, and acute toxicity: In vitro, in vivo, and in silico study. Open Chemistry. 2024;22(1). doi: 10.1515/chem-2023-0214

[48] Luo X, Lim L-T. A colorimetric assay for the detection of monoterpenes in hemp (Cannabis sativa) essential oil. Measurement: Food. 2023;11:100102. doi: 10.1016/j.meafoo.2023.100102

[49] Sut S, Mazzara E, Maggi F, Castagliuolo I, Dall’Acqua S, Petrelli R. *Cannabis sativa* essential oils orally administered to CD1 mice: Tissue distribution of main constituents. Fitoterapia. 2024;178:106147. doi: 10.1016/j.fitote.2024.106147 39094699

[50] Chen H, Gu Z, Yang L, Yang R, Ji Y, Zeng Q, et al. Optimization extraction of rosemary essential oils using hydrodistillation with extraction kinetics analysis. Food Sci Nutr. 2021;9(11):6069–77. doi: 10.1002/fsn3.2549 34760238 PMC8565215

[51] Zheljazkov VD, Cantrell CL, Astatkie T, Jeliazkova E. Distillation time effect on lavender essential oil yield and composition. J Oleo Sci. 2013;62:195–199. doi: 10.5650/jos.62.195 23535305

[52] Semerdjieva IB, Shiwakoti S, Cantrell CL, Zheljazkov VD, Astatkie T, Schlegel V, et al. Hydrodistillation extraction kinetics regression models for essential oil yield and composition in *Juniperus virginiana, J. excelsa, and J. sabina*. Molecules. 2019;24:986. doi: 10.3390/molecules24050986 30862073 PMC6429388

[53] Naz S, Hanif MA, Ansari TM, Al-Sabahi JN. A comparative study on hemp (*Cannabis sativa*) essential oil extraction using traditional and advanced techniques. Guang Pu Xue Yu Guang Pu Fen Xi. 2017;37:306–311. 30221908

[54] Pieracci Y, Ascrizzi R, Terreni V, Pistelli L, Flamini G, Bassolino L, et al. Essential oil of *Cannabis sativa* L: Comparison of yield and chemical composition of 11 hemp genotypes. Molecules. 2021;26:4080. doi: 10.3390/molecules26134080 34279420 PMC8271456

[55] Kusuhara M, Maruyama K, Ishii H, Masuda Y, Sakurai K, Tamai E, et al. A Fragrant Environment Containing α-Pinene Suppresses Tumor Growth in Mice by Modulating the Hypothalamus/Sympathetic Nerve/Leptin Axis and Immune System. Integr Cancer Ther. 2019;18:1534735419845139. doi: 10.1177/1534735419845139 31018712 PMC6484235

[56] Allenspach M, Steuer C. α-Pinene: A never-ending story. Phytochemistry. 2021;190:112857. doi: 10.1016/j.phytochem.2021.112857 34365295

[57] Gölükcü M, Çınar O, Tokgöz H, Uysal Bayar F, Özek T. Effect of Different Drying Methods and Distillation Times on Essential Oil Composition and Antioxidant Content of Rosemary. Akademik Gıda. 2024;22(3):186–94. doi: 10.24323/akademik-gida.1603558

[58] Rivas da Silva AC, Lopes PM, Barros de Azevedo MM, Costa DC, Alviano CS, Alviano DS. Biological activities of α-pinene and β-pinene enantiomers. Molecules. 2012;17:6305–6316. doi: 10.3390/molecules17066305 22634841 PMC6268778

[59] Salehi B, Upadhyay S, Erdogan Orhan I, Kumar Jugran A, L D Jayaweera S, A Dias D, et al. Therapeutic Potential of α- and β-Pinene: A Miracle Gift of Nature. Biomolecules. 2019;9(11):738. doi: 10.3390/biom9110738 31739596 PMC6920849

[60] Feng X, Xiao Z, Yang Y, Chen S, Liao S, Luo H, et al. β-Pinene Derived Products With Enhanced In Vitro Antimicrobial Activity. Natural Product Communications. 2021;16(2). doi: 10.1177/1934578x21992218

[61] Api AM, Belmonte F, Belsito D, Biserta S, Botelho D, Bruze M, et al. RIFM fragrance ingredient safety assessment, myrcene, CAS Registry Number 123-35-3. Food Chem Toxicol. 2020;144:111631. doi: 10.1016/j.fct.2020.111631 32783999

[62] Surendran S, Qassadi F, Surendran G, Lilley D, Heinrich M. Myrcene—What are the potential health benefits of this flavouring and aroma agent? Front Nutr. 2021;8:699666. doi: 10.3389/fnut.2021.699666 34350208 PMC8326332

[63] Wu Z, Li Z, Liang Y. Myrcene exerts anti-tumor effects on oral cancer cells in vitro via induction of apoptosis. Trop J Pharm Res. 2022;21:933–8.

[64] Johnson MB, McKnight S, Taylor EP, Mechtler L, Ralyea CC Jr. The Effects of β-myrcene on Simulated Driving and Divided Attention: A Double-Blind, Placebo-Controlled, Crossover Pilot Study. Cannabis. 2023;6(1):9–19. doi: 10.26828/cannabis/2023.01.002 37287732 PMC10212270

[65] Zheljazkov VD, Astatkie T, Schlegel V. Distillation Time Changes Oregano Essential Oil Yields and Composition but Not the Antioxidant or Antimicrobial Activities. Horts. 2012;47(6):777–84. doi: 10.21273/hortsci.47.6.777

[66] Erasto P, Viljoen AM. Limonene - a Review: Biosynthetic, Ecological and Pharmacological Relevance. Natural Product Communications. 2008;3(7). doi: 10.1177/1934578x0800300728

[67] Mazzara E, Torresi J, Fico G, Papini A, Kulbaka N, Dall’Acqua S, et al. A comprehensive phytochemical analysis of terpenes, polyphenols and cannabinoids, and micromorphological characterization of 9 commercial varieties of *Cannabis sativa* L. Plants. 2022;11:891. doi: 10.3390/plants11070891 35406871 PMC9003298

[68] Chen X, Ding Y, Guan H, Zhou C, He X, Shao Y, et al. The Pharmacological Effects and Potential Applications of Limonene From Citrus Plants: A Review. Natural Product Communications. 2024;19(5). doi: 10.1177/1934578x241254229

[69] Farré-Armengol G, Filella I, Llusià J, Peñuelas J. β-Ocimene, a key floral and foliar volatile involved in multiple interactions between plants and other organisms. Molecules. 2017;22:1148. doi: 10.3390/molecules22071148 28703755 PMC6152128

[70] Oswald IWH, Paryani TR, Sosa ME, Ojeda MA, Altenbernd MR, Grandy JJ, et al. Minor, nonterpenoid volatile compounds drive the aroma differences of exotic *Cannabis*. ACS Omega. 2023;8:39203–39216. doi: 10.1021/acsomega.3c04496 37901519 PMC10601067

[71] Zhekova G, Nedkov N. Quantitative changes in major components of lavender oil during the distillation process. Agric Sci Technol. 2010;2:26–8.

[72] Khaleel C, Tabanca N, Buchbauer G. α-Terpineol, a natural monoterpene: A review of its biological properties. Open Chemistry. 2018;16(1):349–61. doi: 10.1515/chem-2018-0040

[73] Chopra M, Frimpong-Manson K, Wilkerson JL. Evaluation of α-terpineol Attenuation of Precipitated Nicotine Withdrawal Behaviors in Mice. The Journal of Pharmacology and Experimental Therapeutics. 2024;389:104. doi: 10.1124/jpet.104.925870

[74] de Sousa DP, Quintans L Jr, de Almeida RN. Evolution of the Anticonvulsant Activity of α-Terpineol. Pharmaceutical Biology. 2007;45(1):69–70. doi: 10.1080/13880200601028388

[75] Alipour H-R, Yaghmaei P, Ahmadian S, Ghobeh M, Ebrahim-Habibi A. A study on alpha-terpineol in Alzheimer’s disease with the use of rodent in vivo model, restraint stress effect and in vitro Amyloid beta fibrils. Braz J Pharm Sci. 2022;58. doi: 10.1590/s2175-97902022e19090

[76] Yao J, Guo J, Yang Z, Li H, Qiu B. Degradation of α-terpineol in aqueous solution by UV/H2O2: kinetics, transformation products and pathways. Water Sci Technol. 2019;79:2195–2202. doi: 10.2166/wst.2019.221 31318357

[77] Lima NGPB, De Sousa DP, Pimenta FCF, Alves MF, De Souza FS, Macedo RO, et al. Anxiolytic-like activity and GC-MS analysis of (R)-(+)-limonene fragrance, a natural compound found in foods and plants. Pharmacol Biochem Behav. 2013;103(3):450–4. doi: 10.1016/j.pbb.2012.09.005 22995322

[78] Zou JJ, Cai X, Zeng XL, Yang J, Wang CY. Characterization of aroma-active compounds from sweet osmanthus (*Osmanthus fragrans*) by SDE and SPME coupled with GC-MS and GC-olfactometry. Intl J Agric Biol. 2019;22: 277‒82.

[79] Eržen M, Košir IJ, Ocvirk M, Kreft S, Cerenak A. Metabolomic analysis of cannabinoid and essential oil profiles in different hemp (*Cannabis sativa* L.) phenotypes. Plants. 2021;10:966. doi: 10.3390/plants10050966 34066131 PMC8151046

[80] Isidore E, Karim H, Ioannou I. Extraction of phenolic compounds and terpenes from *Cannabis sativa* L. by-products: From conventional to intensified processes. Antioxidants. 2021;10:942. doi: 10.3390/antiox10060942 34200871 PMC8230455

[81] Nagy DU, Cianfaglione K, Maggi F, Sut S, Dall’Acqua S. Chemical characterization of leaves, male and female flowers from spontaneous cannabis (*Cannabis sativa* var. *spontanea*) growing in Hungary. Chem Biodivers. doi: 10.1002/cbdv.201800562 30548994

[82] Tabiś A, Szumny A, Bania J, Pacyga K, Lewandowska K, Kupczyński R. Comparison of the effects of essential oils from *Cannabis sativa* and *Cannabis indica* on selected bacteria, rumen fermentation, and methane production-*In vitro* study. Int J Mol Sci. 2024; 25:5861. doi: 10.3390/ijms25115861 38892045 PMC11172183

[83] Scandiffio R, Geddo F, Cottone E, Querio G, Antoniotti S, Pia Gallo M, et al. Protective effects of (E)-β-caryophyllene (BCP) in chronic inflammation. Nutrients. 2020;12:3273. doi: 10.3390/nu12113273 33114564 PMC7692661

[84] Gertsch J, Leonti M, Raduner S, Racz I, Chen J-Z, Xie X-Q, et al. Beta-caryophyllene is a dietary cannabinoid. Proc Natl Acad Sci U S A. 2008;105(26):9099–104. doi: 10.1073/pnas.0803601105 18574142 PMC2449371

[85] Dalavaye N, Nicholas M, Pillai M, Erridge S, Sodergren MH. The clinical translation of α-humulene – A scoping review. Planta Med. 2024; 90:664–674. doi: 10.1055/a-2307-8183 38626911 PMC11254484

[86] Savoldi TL, Glamoćlija J, Soković M, Gonçalves JE, Ruiz SP, Linde GA, et al. Antimicrobial activity of essential oil from *Psidium cattleianum* Afzel. ex Sabine leaves. Bol Latinoam Caribe Plantas Med Aromát. 2020;19:614–27.

[87] Rusdi NA, Goh HH, Baharum SN. GC-MS/olfactometric characterization and aroma extraction dilution analysis of aroma active compounds in Polygonum minus essential oil. PlantOmics J. 2016;9:289–94.

[88] Pérez-López A, Cirio AT, Rivas-Galindo VM, Aranda RS, de Torres NW. Activity againstStreptococcus pneumoniaeof the Essential Oil and δ-Cadinene Isolated fromSchinus molleFruit. Journal of Essential Oil Research. 2011;23(5):25–8. doi: 10.1080/10412905.2011.9700477

[89] Guo X, Shang X, Li B, Zhou XZ, Wen H, Zhang J. Acaricidal activities of the essential oil from *Rhododendron nivale* Hook. f. and its main compound, δ-cadinene against *Psoroptes cuniculi*. Veterinary Parasitology. 2017;236:51–54. doi: 10.1016/j.vetpar.2017.01.028 28288764

[90] Gong X, Han Y, Zhu J, Hong L, Zhu D, Liu J, et al. Identification of the aroma-active compounds in Longjing tea characterized by odor activity value, gas chromatography- olfactometry, and aroma recombination. International Journal of Food Properties. 2017;20(sup1):S1107–21. doi: 10.1080/10942912.2017.1336719

[91] Mathela CS, Tiwari M, Sammal SS, Chanotiya CS. Valeriana wallichiiDC, a New Chemotype from Northwestern Himalaya. Journal of Essential Oil Research. 2005;17(6):672–5. doi: 10.1080/10412905.2005.9699029

[92] Sen-Utsukarci B, Kessler SM, Akbal-Dagistan O, Estep AS, Tabanca N, Kurkcuoglu M, et al. Chemical composition and biological activities of Valeriana dioscoridis SM. roots. South African Journal of Botany. 2021;141:306–12. doi: 10.1016/j.sajb.2021.05.007

[93] Albuquerque BN de L, Da Silva MFR, Da Silva PCB, De Lira Pimentel CS, Lino Da Rocha SK, Farias De Aguiar JCR de O, et al. Oviposition deterrence, larvicidal activity and docking of β-germacrene-D-4-ol obtained from leaves of Piper corcovadensis (Piperaceae) against Aedes aegypti. Industrial Crops and Products. 2022;182:114830. doi: 10.1016/j.indcrop.2022.114830

[94] Doorandïshan M, Gholamï M, Ebrahïmï P, Jassbï AR. Spathulenol as the most abundant component of essential oil of Moluccella aucheri (Boiss.) Scheen. Natural Volatiles and Essential Oils. 2021;8(2):37–41. doi: 10.37929/nveo.817562

[95] Sánchez-Mendoza ME, Cruz-Antonio L, Cupido-Sánchez MG, Castillo GG, Arrieta J. Gastroprotective activity of caryophyllene oxide: the role of nitric oxide, prostaglandins and sulfhydryls. J Appl Pharm Sci. 2014;4:001–5. doi: 10.7324/JAPS.2014.40901

[96] Tan M, Zhou L, Huang Y, Wang Y, Hao X, Wang J. Antimicrobial activity of globulol isolated from the fruits of *Eucalyptus globulus* Labill. Nat Prod Res. 2008; 22:569–575. doi: 10.1080/14786410701592745 18569693

[97] dos Santos Cavalcanti A, de Souza Alves M, da Silva LCP, dos Santos Patrocínio D, Sanches MN, Chaves DS de A, et al. Volatiles composition and extraction kinetics from Schinus terebinthifolius and Schinus molle leaves and fruit. Revista Brasileira de Farmacognosia. 2015;25(4):356–62. doi: 10.1016/j.bjp.2015.07.003

[98] Janatová A, Doskočil I, Božik M, Fraňková A, Tlustoš P, Klouček P. The chemical composition of ethanolic extracts from six genotypes of medical cannabis (*Cannabis sativa* L.) and their selective cytotoxic activity. Chem Biol Interact. 2022;353:109800. doi: 10.1016/j.cbi.2022.109800 34995571

[99] Baldwin TA, Oberbauer SF. Essential oil content of Rhododendron tomentosum responds strongly to manipulation of ecosystem resources in Arctic Alaska. Arctic Science. 2022;8(3):916–34. doi: 10.1139/as-2020-0055

[100] Bomfim DS, Ferraz RP, Carvalho NC, Soares MB, Pinheiro ML, Costa EV, et al. Eudesmol isomers induce caspase-mediated apoptosis in human hepatocellular carcinoma HepG2 cells. Basic Clin Pharmacol Toxicol. 2013;113:300–6. doi: 10.1111/bcpt.12097 23786320

[101] Uchida T, Matsubara Y, Koyama Y. Structures of two novel sesquiterpenoids formed by the lead tetraacetate oxidation of β-caryophyllene. Agric Biol Chem. 1989;53:3011–5.

[102] Almeida PdV, Tolouei SEL, Minteguiaga M, Chaves DSA, Heiden G, Khan SI, et al. Chemical profiles and cytotoxic activities of essential oils from six species of *Baccharis* subgenus *Coridifoliae* (Asteraceae). Chem Biodivers. 2023;20:e202300862. doi: 10.1002/cbdv.202300862 37647349

[103] Acimovic M, Pezo L, Stankovic-Jeremic J, Todosijevic M, Rat M, Tesevic V, et al. The quantitative structure-retention relationship of the GC-MS profile of yarrow essential oil. Acta per tech. 2021;(52):123–32. doi: 10.2298/apt2152123a

[104] Ben Sghaier M, Mousslim M, Pagano A, Ammari Y, Luis J, Kovacic H. β-eudesmol, a sesquiterpene from Teucrium ramosissimum, inhibits superoxide production, proliferation, adhesion and migration of human tumor cell. Environ Toxicol Pharmacol. 2016;46:227–33. doi: 10.1016/j.etap.2016.07.019 27497729

[105] Asakura K, Kanemasa T, Minagawa K, Kagawa K, Yagami T, Nakajima M, et al. α-Eudesmol, a P/Q-type Ca^2^+ channel blocker, inhibits neurogenic vasodilation and extravasation following electrical stimulation of trigeminal ganglion. Brain Res.2000; 873:94–101. doi: 10.1016/s0006-8993(00)02527-0 10915814

[106] Rodilla JM, Silva LA, Martinez N, Lorenzo D, Davyt D, Castillo L. Advances in the identification and agrochemical importance of sesquiterpenoids from Bulnesia sarmientoi essential oil. Ind Crop Prod. 2011;33:497–503.

[107] Gao X, Zhang Y, Wang W, Zhang Z, Li C, Lou H. α-Bisabolol exerts anti-inflammatory action and ameliorates collagen-induced arthritis in rats. Indian J Anim Res. 2022;56:1010–6.

[108] Murata Y, Kokuryo T, Yokoyama Y, Yamaguchi J, Miwa T, Shibuya M, et al. The anticancer effects of novel α-bisabolol derivatives against pancreatic cancer. Anticancer Res. 2017;37:589–598. doi: 10.21873/anticanres.11352 28179305

[109] Nance MR, Setzer WN. Volatile components of aroma hops (Humulus lupulus L.) commonly used in beer brewing. J Brew Distilling. 2011;2:16–22.

[110] Maggini V, Calvi L, Pelagatti T, Gallo ER, Civati C, Privitera C, et al. An Optimized Terpene Profile for a New Medical Cannabis Oil. Pharmaceutics. 2022;14(2):298. doi: 10.3390/pharmaceutics14020298 35214031 PMC8879232

[111] Spinozzi E, Boldrini L, Ferrati M, Betti E, Ricciutelli M, Zheljazkov VD, et al. Waste from hemp essential oil production: How distillation methods shape the byproducts value. Industrial Crops and Products. 2025;230:121094. doi: 10.1016/j.indcrop.2025.121094

[112] Bardhi K, Coates S, Watson CJW, Lazarus P. Cannabinoids and drug metabolizing enzymes: potential for drug-drug interactions and implications for drug safety and efficacy. Expert Rev Clin Pharmacol. 2022;15:1443–1460. doi: 10.1080/17512433.2022.2148655 36384377

[113] Martinez AS, Lanaridi O, Stagel K, Halbwirth H, Schnürch M, Bica-Schröder K. Extraction techniques for bioactive compounds of cannabis. Nat Prod Rep. 2023;40:676–717. doi: 10.1039/D2NP00059H 36625451

[114] Fućak T, Kreft S, Svedružić ŽM, Tavčar E. Mechanism and kinetics of CBDA decarboxylation into CBD in hemp. J Plant Biochem Biotechnol. 2023;32(3):608–21. doi: 10.1007/s13562-023-00847-z

[115] Zheljazkov VD, Noller JS, Dale R, Maggi F. Terpenes and cannabinoids yields and profile from direct-seeded and transplanted CBD-*Cannabis sativa*. J Agric Food Chem. 2022; 70:10417–10428. doi: 10.1021/acs.jafc.1c06912 35436102

